# Magnetic Internal Corrosion Detection Sensor for Exposed Oil Storage Tanks

**DOI:** 10.3390/s21072457

**Published:** 2021-04-02

**Authors:** Ahmad Aljarah, Nader Vahdati, Haider Butt

**Affiliations:** Department of Mechanical Engineering, Khalifa University of Science and Technology, SAN Campus, Abu Dhabi P.O. Box 127788, United Arab Emirates; ahmad.aljarah@ku.ac.ae (A.A.); haider.butt@ku.ac.ae (H.B.)

**Keywords:** corrosion detection sensor, electromagnetic numerical analysis, internal corrosion, oil storage tanks, FEA

## Abstract

Corrosion in the oil and gas industry represents one of the major problems that affect oil production and transportation processes. Several corrosion-inspection technologies are in the market to detect internal and external corrosion of oil storage tanks, but inspection of storage tanks occurs every 3 to 7 years. In between inspection interval, aggressive corrosion can potentially occur, which makes the oil and gas industry vulnerable to accidents. This study proposes a new internal corrosion detection sensor based on the magnetic interaction between a rare-earth permanent magnet and the ferromagnetic nature of steel, used to manufacture oil storage tanks. Finite element analysis (FEA) software was used to analyze the effect of various sensor parameters on the attractive force between the magnet and the steel. The corrosion detection sensor is designed based on the FEA results. The experimental testing of the sensor shows that it is capable of detecting internal metal loss due to corrosion in oil storage tanks within approximately 8 mm of the internal surface thickness. The sensor showed more than two-fold improvement in the detection range compared to previous sensor proposed by the authors. Furthermore, the sensor of this paper provides a monitoring rather than occasional inspection solution.

## 1. Introduction

The oil and gas (O & G) industry is one of the largest industries in the world, which has evolved significantly in the past couple of centuries and was the main factor that brought and enhanced the industrial revolution, which was the base that all of our current advanced technologies are built on. Running and managing such a huge industry requires a lot of work and concentration to keep all the facilities with different sectors under control, avoiding any problems that may affect oil and gas production and transportation processes. Correctly operating the manufacturing and maintenance of these facilities with the minimum possible cost guarantees achieving great profits.

One of the major problems that should be carefully studied when designing and operating all stages of O & G production, is corrosion, especially since most oil and gas reserves exist in challenging locations and environments such as remote arctic locations or hard-to-manage reservoirs with dispersed sand accompanied with a high content of CO_2_ or H_2_S as well as high pressures and temperatures, which leads to material deterioration [1].

Corrosion is a dangerous and costly problem that not only affects the O & G industry but also affects almost all industries and public infrastructures. The reliability of such infrastructures contributes to the industrial productivity and economic competitiveness. Infrastructures such as highways, bridges, airports, buildings, water and energy supply, and power generation systems are all part of huge systems that require continuous corrosion maintenance and attention. Corrosion takes place on different engineering materials when they become exposed to various environmental conditions such as chemicals, stress, radiation, water, oxygen, temperature, salt, etc. [2]. These environmental conditions differ depending on the engineering application, the type of industry, and the location of the facility. For example, atmospheric corrosion that affects historical buildings (metal structures) was studied in [3] in order to develop a time-dependent model that can predict corrosion propagation in wrought iron structures. The authors built their results based on two environmental conditions (marine and urban–industrial). The corrosion-predictive models that the authors used accounted for many variables such as exposure time, concentration of atmospheric gas, relative humidity, ambient temperature, and amount of rainfall.

As for corrosion in the O & G industry, which is the focus of this paper, Section 2.1, “Overview of Corrosion in the Oil and Gas Industry” specifically elaborates on the corrosion environment near oil and gas storage tanks and pipelines.

The oil and gas production process occurs in different stages starting from extraction in wells locations to the stages where it is used as fuel. These stages are production, transmission through pipelines, transportation in tanks, storage, refining, and distribution. Throughout the process, various types of materials are used including metals representing the vast majority and non-metals. Due to its low cost, availability, and ease of fabrication, carbon steel is the main material that is used in at least 80% of all components in the O & G industry. On the other hand, carbon steel is prone to one of the major material deterioration problems, which is corrosion [4].

Corrosion problems cost the O & G companies billions of dollars around the world every year. Different corrosion detection, mitigation, and prevention methods are presented in Section 2, each one of them is based on a different technology, from magnetic flux leakage (MFL) devices to ultrasonic and others.

In this study, a new sensor to detect internal corrosion of oil storage tanks will be presented. This new sensor sits outside of the oil storage tank without a need for any modifications to the storage tank, and it can detect change in the oil storage tank wall thickness (metal loss) due to corrosion. This paper documents the design and analysis of this new sensor plus its experimental results.

## 2. Literature Review

### 2.1. Overview of Corrosion in the Oil and Gas Industry

Corrosion can be simply defined as the deterioration of metal surfaces due to the interaction with a nearby environment. In the O & G industry, these metal surfaces are mainly the walls of the pipelines and storage tanks that transport, carry, and store oil and gas products. The interaction with the nearby environment usually occurs in the form of an electromechanical reaction that involves electron transfer in an electrolyte between an anode and a cathode in the presence of aqueous media such as soil and water [5,6].

The corrosion environment in the O & G industry mainly consists of three main constituents: carbon dioxide (CO_2_), hydrogen sulfide (H_2_S), and water. Each type of corrosion that is related to one of these environments has a unique chemical reaction. When carbon dioxide (CO_2_), for example, reacts with iron (Fe) and water as the aqueous media, it produces iron carbonate (FeCO_3_) and hydrogen (H_2_), which is known as sweet corrosion. Iron sulfide (FeSx) is another corrosion product that is formed when hydrogen sulfide (H_2_S) reacts with iron (Fe) [6].

In the oil production process, which consists of three main stages: primary, secondary, and tertiary, oil is extracted from underground reservoirs using different mechanisms. In the case of primary oil recovery, the natural drive energy of the reservoir (the reservoir’s high pressures) will bring the oil to the surface, but if this natural drive energy is not sufficient, sucker-rod lift (beam pumping) may be used or water and gas injection are employed to drive the oil to the surface. Carbon dioxide (CO_2_) gas, one of the commonly used gases, is normally injected into a reservoir to bring the oil to the surface and enhance the oil recovery. Large amounts of CO_2_ are injected into the reservoirs, which significantly enhances the oil mobility, thus, increasing oil production [7], but the CO_2_ gases mixing with water form carbonic acid (H_2_CO_3_), and the carbonic acid will react with iron (interior surfaces of the oil storage tanks and pipelines), forming iron carbonate (FeCO_3_) and hydrogen (H_2_).

As for hydrogen sulfide, the oil reservoirs act as a main source for hydrogen sulfide (H_2_S), since it is produced as a result of microbial breakdown of organic material. Hydrogen sulfide can then react with the interior surfaces of the oil storage tanks and pipelines (iron) and form iron sulfide (FeSx).

Corrosion of oil pipelines and storage tanks occurs in different forms, such as uniform or general metal loss, pitting corrosion, erosion–corrosion, stray current corrosion, and microbiologically influenced corrosion (MIC). Each type of these forms usually occurs due to different causes, and there are several ways to develop preventive mechanisms for each one. For example, uniform metal loss usually occurs along the internal and/or external surfaces of pipelines and storage tanks, and it can be mitigated for the external surface by using surface coating and for the internal surface by surface coating, thermoplastic liners, or proper material selection. On the other hand, pitting corrosion affects specific spots on the metal surface and usually occurs due to material defects, mechanical damages, or scratches on the surface. It may be prevented by using proper material according to the surrounding environment [5] and better surface finish.

Multiple corrosion detection techniques are used in the industry. According to [8], they can be divided into two main categories: direct and indirect corrosion sensing. Direct corrosion detection methods mainly aim for detecting the rate or process of corrosion itself, whereas indirect corrosion sensing mechanisms study either the environmental causes behind the corrosion process, such as the pH levels, water, and CO_2_ levels, or the consequences that are caused by corrosion, such as the change in wall thickness or strain values.

### 2.2. Corrosion-Inspection Technologies in the Oil and Gas Industry

Despite the multiplicity of preventing techniques for the different types of corrosion, it is still a big problem that costs the O & G companies billions of dollars around the world every year. The global annual cost of corrosion in the O & G industry is estimated as USD 1.372 billion, and it is increasing every year [9]. Hence, companies have invested in the development of corrosion-inspection technologies in order to detect early stages of corrosion and preventing leakage incidents or rupture of oil pipelines and storage tanks.

**Figure 1:**
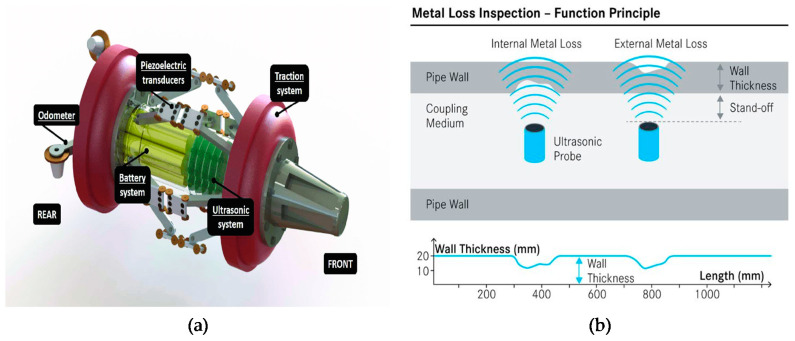
(**a**) Pipeline inspection gauge (PIG) with ultrasonic system attached on its surface (production-ready design) [8]. (**b**) Ultrasonic sensor working principle [10].

Pipeline inspection gauges (PIGs) are one of the most commonly used methods to inspection oil pipelines and storage tanks for corrosion. In the case of pipelines, several PIGs are first used to clean the interior pipeline surface before the pipeline is inspected for metal loss due to corrosion, defects, and cracks. The PIG device shown in Figure 1a travels inside the pipeline, driven by the fluid pressure.

The two main internal inspection technologies, used by manufacturers of smart PIGs, are magnetic flux leakage (MFL) and ultrasonic (UT), and these two technologies may incorporate spring loaded arms with rolling wheels that ride on the inner surface of the pipeline, looking for dents and depressions.

Electromagnetic acoustic transducer (EMAT) or ultrasonic (UT) PIGs use either an electromagnetic transducer or a piezo-electric transducer to induce ultrasonic waves within the steel. The sensor emits an ultrasonic beam that is reflected from the external and internal surfaces of the pipeline and any geometrical irregularities’ locations. When the ultrasonic wave hits a crack or any abnormality, the sound wave is reflected back, and those reflected waves can indicate cracks or dents or metal loss due to corrosion [8]. Figure 1a shows an ultrasonic system attached to a PIG.

Magnetic flux leakage (MFL) technology uses a strong magnetic field in the pipeline wall, through magnets circumferentially located around the PIG. The wall of the pipeline is magnetized until saturation. In addition to the magnets, there are sensors placed between the magnets that record any disturbance in the magnetic field, which indicates metal loss. The resulting magnetic field is then analyzed in order to detect any abnormalities on the external or internal surface of the pipeline wall. The defects can then be detected by studying the change in the magnetic flux lines. For the areas of defects or cracks, the flux lines will be bent or escape the material surface [11,12]. Although this method is widely used in the industry, it cannot distinguish whether the defects detected are on the external or internal surface of the pipeline unless support vector machines (SVM) are used to do the classification [12,13].

**Figure 2:**
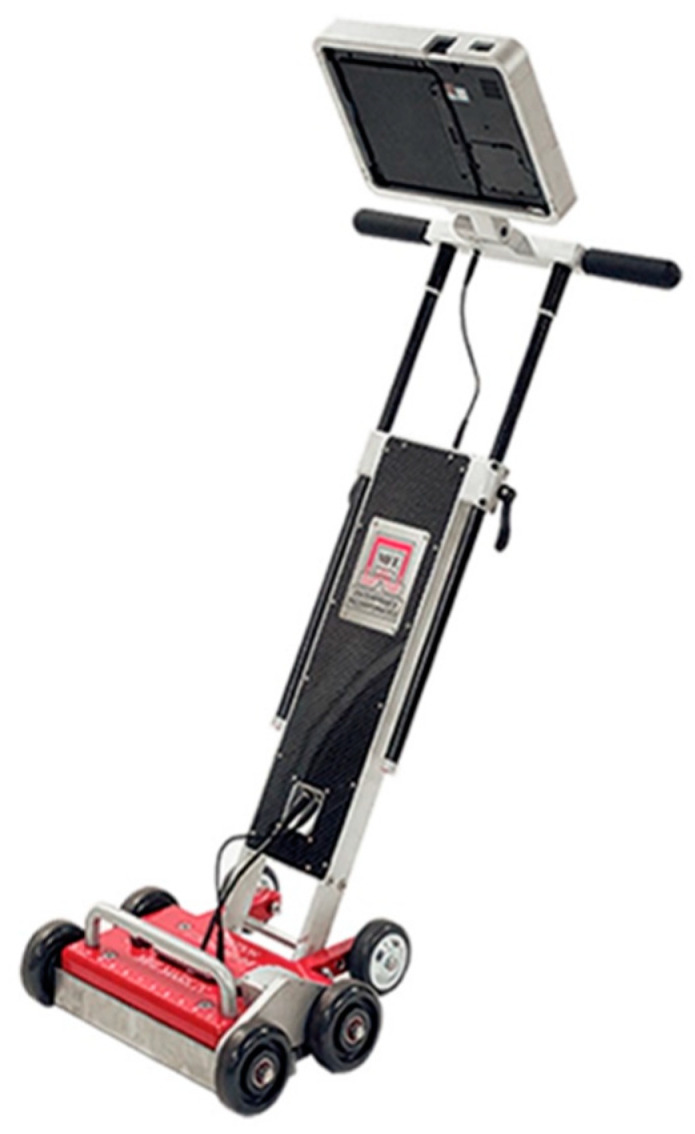
Typical oil storage tank floor scanner using magnetic flux leakage (MFL) technology (production-ready design).

The above two PIGs can collect various data indicating metal loss due to corrosion, cracks, dents, and other irregularities on pipelines walls [14], but it is important to mention that even though PIGs are very effective, they are only inspection tools, and they are by no means health monitoring tools, capable of providing real-time corrosion rate data. The same MFL and UT technologies that are used to inspect oil pipelines are also used for the inspection of storage tanks. Figure 2 shows a typical tank floor scanner using MFL technology to inspect oil storage tank bottom floor.

Although oil storage tank floor scanners using MFL technology (similar to pipeline inspection gauge (PIG) technology) are widely used for corrosion detection of oil storage tanks, they are only used for inspection purposes every 5–7 years [15] or per the company policy, with some oil companies using the MFL-based scanners every 3 to 5 years. The reason behind this low-frequency usage of scanners is mainly the cost of the operation. Furthermore, scanners are used as inspection tools and not as health monitoring tools, meaning they do not provide continuous corrosion monitoring. Aggressive internal corrosion can occur in between the inspection intervals, and the oil companies may be unaware of it. Even when regular inspection of the oil tanks occurs, there has been cases where some service providers, after inspection, have given oil companies assurance that the oil tank is fit for service, but later on, accidents have occurred due to the service provider not possessing the technology to detect threshold wall thickness necessary to continue operation.

In regard to magnetic flux leakage technique, one of its usage limitations, especially on thick wall pipelines, is that magnetic saturation of pipeline wall material is required [12]. It is also limited to near-surface defects detection and needs some data processing to differentiate between external and internal defects.

Ultrasonic detection devices are not commonly used for inspection purposes because their accuracy can be easily affected by debris and mud on the pipeline’s wall surfaces; hence, the pipeline must be carefully cleaned before using the UT device. Furthermore, the ultrasonic inspection method is not very sensitive in detecting small cracks or pitting corrosion. Another issue is that ultrasonic PIGs require a medium to work, meaning the pipeline or the storage tank needs to be filled with some liquid, such as seawater or water. Emptying and partial or completely filling the oil storage tank or pipelines with another liquid, in order to do ultrasonic inspection, impacts production, so this is why this technology is less used in the O & G industry.

Gloria et al. [11] developed an internal corrosion detection sensor that has the ability to do the inspection without the necessity of achieving magnetic saturation for the pipeline wall material, but it was mainly applied to thick pipelines with a small diameter. The idea of this sensor is based on a direct magnetic response from a small area of the wall where the distortion of the magnetic flux lines caused by metal loss can be detected using a stem that passes over the defect. This procedure can also predict the depth of the defect.

Hydrostatic testing is another inspection method used by the oil industry for inspection of pipelines and storage tanks. It involves isolating a portion of the pipeline using valves and fittings, filling it out with water or sea water, and then, pressurizing the line to a specified pressure to check for leaks. For exposed above the ground pipelines, the pipeline is pressurized for a minimum of four continuous hours with a pressure of near 125% of the maximum allowable operating pressure (MAOP). The pressure is monitored using a pressure transducer. The pipeline must not be over-pressurized, as this pressure is applied to not only the pipe, but also to the valves, and the fittings and the high pressure may cause failures to these components or even fracture the pipeline. If the pipeline cracks due to the pressure, or there was already a small leak, then there will be a pressure loss, and the location of the cracks or leaks can be visually identified using a dye added to the water [16]. The cracks are then repaired, and after the repair, the pipeline is placed back into service. Hydrostatic testing is not only used to check for leaks in pipelines but also used for detection of leaks in oil storage tanks. It is important to mention that hydrostatic testing is time consuming and very costly, and it does not really shed light on the state of the corrosion of the pipeline or the storage tank. Furthermore, the method interferes with the oil and gas transportation operations and can be destructive if the storage tank or the pipes are over-pressurized. Furthermore, the large amounts of water used in this process need to be specially treated afterward due to the toxic materials present in the oil storage tanks or the oil and gas pipelines.

An optical inspection system is a corrosion and defects inspection technique that was designed by [17] in order to perform internal pipe inspection. The system consists of a calibrated CCD camera, a light ring pattern generator using a 3-axis scanner, and a laser diode that expands the laser beam into the light ring. The CCD camera and the laser diode are positioned in the center of the pipe projecting a ring of light on a specific circular pipe section that is to be inspected; where the rings of light are projected by the laser diode and collected by the CCD camera. The location of the section being investigated is measured with respect to the laser diode location. Furthermore, the illuminated area (cross-sectional area being inspected) is calculated using a defined set of equations. The data collected are then analyzed using image processing where the defects and corroded areas can be identified by analyzing the intensity of the projected light rings; the laser light will be scattered at the defect’s locations changing the intensity levels in the image. This technology can also be used for the inspection of storage tanks for corrosion.

Multiple modifications (drill holes) may have to be made to the pipeline or the storage tank that needs the inspection if optical inspection systems and galvanic sensors are to be used to monitor the internal corrosion. The modifications to the storage tank and pipelines needed to install the optical inspection systems and the galvanic sensors will interfere with oil and gas transportation and production. Furthermore, optical inspection requires excellent knowledge in laser reflectance phenomena, because the laser intensity is very sensitive to the scattering surface smoothness and the angle of incidence, which means that any small mistake may lead to false defect detection as well as the data acquired from CCD cameras requiring careful processing. Additionally, optical inspection systems and galvanic sensors require a power source, but the electricity near oil and gas can potentially cause fires and explosions, if one is not careful.

An interesting work was presented by [18] where the dissolved oxygen level of a 50 m long pipeline inlet was compared with the pipeline outlet, and the decrease in the dissolved oxygen level was attributed to pipeline internal corrosion. A copper–zinc (pure zinc and 99% pure copper) galvanic sensor was inserted into the 50 m long pipeline to reach to the electrolyte solution to measure current. Back-propagation artificial neural network (BP-ANN) was used to estimate the concentration of dissolved oxygen (DO) of the electrolyte according to the measured factors such as the electric current of the galvanic sensor, flow rate, and temperature of the internal electrolyte. It is important to mention that if this sensor is to be used for internal corrosion detection, it will require creating a hole in the pipeline or storage tank in order to attach the galvanic sensor to the pipeline or the storage tank.

Another setup that studies environmental corrosion and corrosion rates in the O & G industry using FBG optical fibers or electrical strain gauges was investigated in [19]. A pre-stressed beam structure was tested experimentally and numerically in order to develop a relationship that detects the strain observed at any point through the thickness of a beam cross-section, which leads to detecting thickness change in a mild steel specimen due to corrosion reaction. The authors concluded that implementing this technique using electrical strain gauges in the oil and gas industry is not suitable due to safety standards. FBG sensors on the other hand provide a better alternative.

Fiber-optics-based sensors are gaining popularity due to being lightweight, being able to provide distributed temperature, pressure, and strain sensing, being able to provide multiplexing on a single fiber, their intrinsic safety (very safe near flammable liquids and gases), and being immune to electromagnetic interference [20]. Due to these mentioned benefits, fiber-optics-based corrosion detection sensors are being researched by many researchers for the O & G industry for corrosion detection and structural health monitoring purposes. Fiber optics sensors can be divided into four different types according to their spatial distribution of the measurements: point, integrated, quasi distributed, and distributed sensors. Point sensors, for example, are used to detect corrosion at only one location, which means that each sensor detects only one point, whereas distributed sensors can be used to detect corrosion continuously across the whole optical fiber [21].

Mohanty et al. [22] designed a plastic optical fiber (POF) sensor to detect structural defects in metallic surfaces by collecting the scattered light reflected by the surface imperfections. The sensor consists of two plastic optical fibers, a photodetector, and an inspection mirror. One of the POFs works as a light source, and the other is connected to the photodetector in order for the reflected beam to be examined. The mirror is placed near the fiber’s tips to increase the voltage of the recorded signal. Thus, corrosion that occurs inside the pipe can be detected by observing the measured voltage intensity where the corroded locations appear as peaks and dips in the voltage. In this paper, pipelines are referred to as application for this work, but this sensor was never tested in a pipeline. When crude oil is present, it is very doubtful that reference [22]’s technology can be used.

Another fiber-optics-based corrosion detection sensor is presented in [23], where the authors introduced a theoretical model based on the contact between a hydrocarbon sensitive polymer cladding of the fiber optics and the hydrocarbon material in the surrounding medium of the pipeline. The design proposes coating the fiber with a material similar to the pipeline material and laying it close to the bottom surface of the oil pipeline so that the coated optical fiber experiences the same flow regime and corrosion rate as the inner bottom surface of the pipeline. When the mild steel coating on the optical fiber is gone due to corrosion, the hydrocarbon sensitive polymer cladding of the fiber is now exposed to hydrocarbons. Hydrocarbons in the surrounding medium will diffuse into the polymer cladding, thus, changing its refractive index (RI) and causing a loss in intensity of a traveling light pulse. The corrosion detection sensor proposed in this paper was a novel work but inserting the fiber to the pipeline and withdrawing the other end of the fiber from the pipeline required two holes, thus, requiring modification to the existing pipeline.

A distributed fiber optics sensor was investigated by Mirzaei et al. [24]. The sensor was designed to detect oil leakage in transportation pipelines based on distributed temperature measurement. Optical fiber cables were placed underneath and near the oil pipeline but not attached to the pipeline. When leakage occurred due to cracks in the pipeline surface, hot oil or gas flowed towards the fiber cable, resulting in a temperature change in the optical fiber. The position of the crack was determined by calculating the time difference between the input and the scattered laser pulses of the fiber. A similar analysis based on distributed temperature sensing (DTS) supported with a finite element numerical analysis was performed by Zhou et al. [25]. One more leak detection sensor using distributed fiber optics was presented by Buerck et al. [26]. Similar to the previous sensor, it detected leak locations by measuring the time difference between short light pulses in the fiber and backscatter signals caused by chemical leakage, but in this configuration, the fiber optics used were made of polymer-clad silica adapted to an optical time-domain reflectometer (OTDF) that is sensitive to liquid hydrocarbons (HC) and hydrocarbon solutions.

An internal corrosion monitoring technique based on distributed strain measurement using optical frequency domain reflectometer (OFDR) technology was proposed by [27]. The sensor was tested to detect and locate uniform and local corrosion in pipelines based on the reduction that occurs to the pipeline wall thickness when it corrodes. The sensor was installed by bonding the optical fiber(s) along the outer circumference of the steel pipe after cleaning and polishing the surface. The job of the optical fiber, attached to the pipeline, was to measure pipeline hoop strain. In [27], it was postulated that the reduction in pipeline wall thickness can be obtained by monitoring the change in the pipeline hoop strain. The problem is that the hoop strain can also change due to changes in the pipeline temperature or internal pressure. It is very difficult to tell if the change in pipeline hoop strain is due to corrosion or due to change in temperature or pressure of the pipeline.

A corrosivity sensor was developed by [28,29], consisting of an array of rectangular sacrificial thin strips of the same planar dimensions but different thicknesses, made of the same metal as that of the pipe. Each metal strip is connected to a light-emitting diode (LED) and constitutes a branch of the sensor circuit. In this paper, four metal strips and LEDs were used, thus, four electrical circuit branches, arranged in parallel. When environment corrosivity is high, it consumes the thinnest metal strip first until the metal strip finally breaks, resulting in a discontinuity in the corresponding branch of the circuit. The LED that was connected to that circuit branch, no longer emits light. The sensor provides a visual indication of severity of the environment corrosivity when all the four LEDs are off. In this paper, the focus was on developing a corrosivity sensor and not on developing an internal corrosion detection sensor.

In reference [30], an optical fiber with an FBG sensor was used to detect corrosion of two rebars embedded in a concrete structure. As the rebars corroded, a wavelength shift was detected by the optical fibers.

Pacheco et al. [31] developed an internal corrosion detection sensor using a permanent magnet and an optical fiber with one FBG sensor. A small gap existed between the magnet and the steel surface. The magnet was attached to one end of the optical fiber, and the other end of the optical fiber was glued to a metal support. The magnet placed the optical fiber in tension due to attractive force between the magnet and the steel surface. As the steel plate corroded, the strain (or force) in the optical fiber reduced, and the change in strain was used to determine the change in plate thickness. The issue with the sensor proposed by [31] is that if larger and stronger magnets are used, the optical fiber will not be able to support those high tensions. It will break. Additionally, the gap between the magnet and the steel surface cannot be maintained. As the tension in the fiber goes down due to steel plate corroding, the air gap between the magnet and the steel surface changes. Furthermore, the sensor was only able to detect corrosion in a steel plate in a submillimeter range, which does not provide an early warning of oil storage tank corrosion status to avoid accidents.

An internal corrosion detection sensor for oil pipelines, based on a similar concept of [31] was investigated by [32]. The sensor of [32] consists of a 25.4 mm (1-inch) diameter and 25.4 mm (1-inch) height samarium–cobalt magnet and a cantilever beam. The magnet was attached to the free end of the cantilever beam. As the sensor was placed on a steel plate, the magnet moved closer to the steel plate due to the attractive force between the magnet and the steel plate. The attractive force deflected the cantilever beam downwards, thus, creating strain in the beam. The strain of the cantilever beam was measured by a strain gauge and an optical fiber. When metal loss occurred in the steel plate, the attractive force between the magnet and the steel plate changed, thus, the cantilever beam strain. The change in the cantilever beam strain was used to detect pipeline corrosion.

The sensor proposed in [32] is an earlier work of this paper’s authors and that sensor had two major drawbacks. The sensor of [32] was only able to detect metal loss, when the steel plate thickness was 3 mm or below. Additionally, the air gap in between the magnet and the steel plate was not controllable and it would vary with time. As the attractive force between the magnet and steel plate would become lower with time due to steel plate corrosion, the air gap would become larger and larger with time, thus, making the sensor ineffective in detecting corrosion.

The corrosion detection sensor design proposed in this paper is based on a similar concept of [32], but with new design modifications to increase the range of wall thickness detection, to precisely control the air gap between the magnet and the steel surface, and to provide a suitable sensor design that can be easily installed on the oil storage tanks without any need for any holes or special fixtures.

## 3. Working Principle of Our Proposed Sensor—Schematic Design

**Figure 3:**
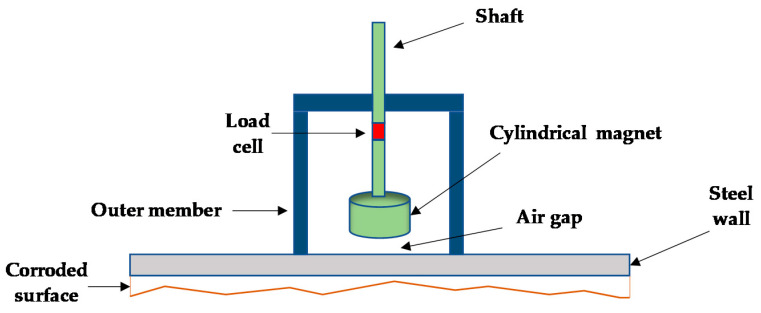
Schematic design of the proposed corrosion detection sensor.

Our proposed corrosion detection sensor consists of a rare-earth magnet (shown in Figure 3 as a cylindrical magnet), a load cell, an inner metal member (shown in green color) holding the magnet, and an outer metal member shown in dark blue color (see Figure 3). There is an air gap between the rare-earth magnet and the steel plate, representing an oil storage tank. The shaft of the inner member is threaded so that the magnet can be moved up or down to adjust the air gap. The attractive force between the rare-earth magnet and the steel plate is measured by the load cell, shown in red color. As the internal side of the steel plate (representing an oil storage tank) starts to corrode, since there is a change in metal thickness and magnetic material property, the attractive force, measured by the load cell, reduces; thus, one can detect the onset of oil storage tank corrosion and progression of the corrosion. The corrosion detection sensor can stick to the steel plate (in our application an oil storage tank) due to presence of the rare-earth magnet without a need for any bolts or screws. To eliminate crevice or galvanic corrosion between the outer member (shown in blue color) and the steel plate (shown in gray color), the outer member and the steel surface can both be coated with high-temperature epoxy or paint to avoid metal-to-metal contact.

## 4. Preliminary Analysis

As explained above, the working principle of the above sensor is based on the magnetic attractive force between the rare-earth magnet and the steel surface of the oil storage tank or an oil pipeline. The attractive force decreases when the thickness of the internal surface of the storage tank wall decreases due to corrosion. In order to provide an estimate for the values of this attractive force and its relationship with the metal thickness loss that occurs in the corroded steel surface, various factors and parameters that can affect this attractive force are numerically analyzed using ANSYS finite element analysis (FEA). These parameters are the magnet size (height, diameter, and magnet material), the separation distance (air gap) between the magnet and the steel wall, and the surface area of the steel wall exposed to the magnet surface. If the effect of each one of the mentioned parameters on attractive force is studied (parametric study), a selection of fixed value for each parameter can be found to optimize the corrosion detection sensor final design and detection range of the sensor and minimize the resulting detection error.

**Figure 4:**
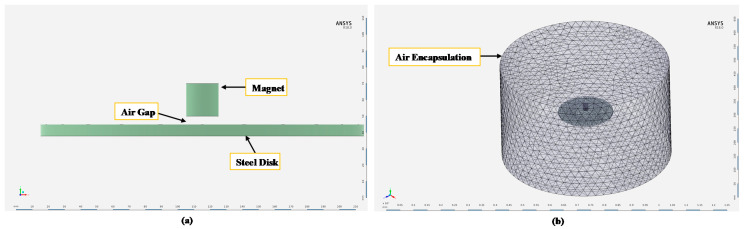
The initial FE model generated in ANSYS. (**a**) FE model components. (**b**) All the sensor components are encapsulated within air encapsulation to generate the mesh.

Figure 4 represents the initial FE model generated in ANSYS to conduct the preliminary analysis needed to optimize the sensor design. For the preliminary analysis, the size of the rare-earth magnet was chosen to be the same as the magnet size of [32].

To start the simulation analysis, an initial value for each parameter was chosen. The baseline parameters, coming from our previous work in [32], are then varied one at a time, and then, the result of each parameter variation on attractive force is studied/analyzed. The initial (baseline) parameters values are a cylindrical magnet with 20 mm height and 20 mm diameter [32], a separation distance of 0.5 mm, and a disk underneath the magnet with 200 mm diameter and 7 mm thickness representing the steel wall of the oil storage tank. The steel disk underneath the magnet, representing the oil storage tank or an oil and gas pipeline, is modeled as 1010 steel [32] with 1010 steel B-H curve property.

### 4.1. Parametric Studies

#### 4.1.1. Magnet Size

**Figure 5:**
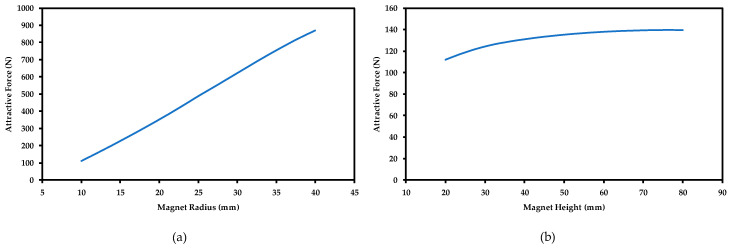
The effect of the magnet size on the attractive (magnetic) force. (**a**) Effect of magnet diameter. (**b**) Effect of magnet height.

The size of the rare-earth magnet is represented by both the height and diameter of the cylindrical magnet. In order to study the effect of each one of these parameters on attractive force, both values are changed individually from 20 mm up to 80 mm. Figure 5 illustrates the results.

The results show that the magnet diameter has a huge impact on the attractive force generated with magnet height kept constant. The attractive force increased by more than 750 N when the magnet diameter was increased, whereas the attractive force has only increased by about 30 N when the height is increased by the same amount. Experimental and numerical data suggest that once the height of the magnet is the same as its diameter, any increase in height only increases the attractive force by a small amount.

**Figure 6:**
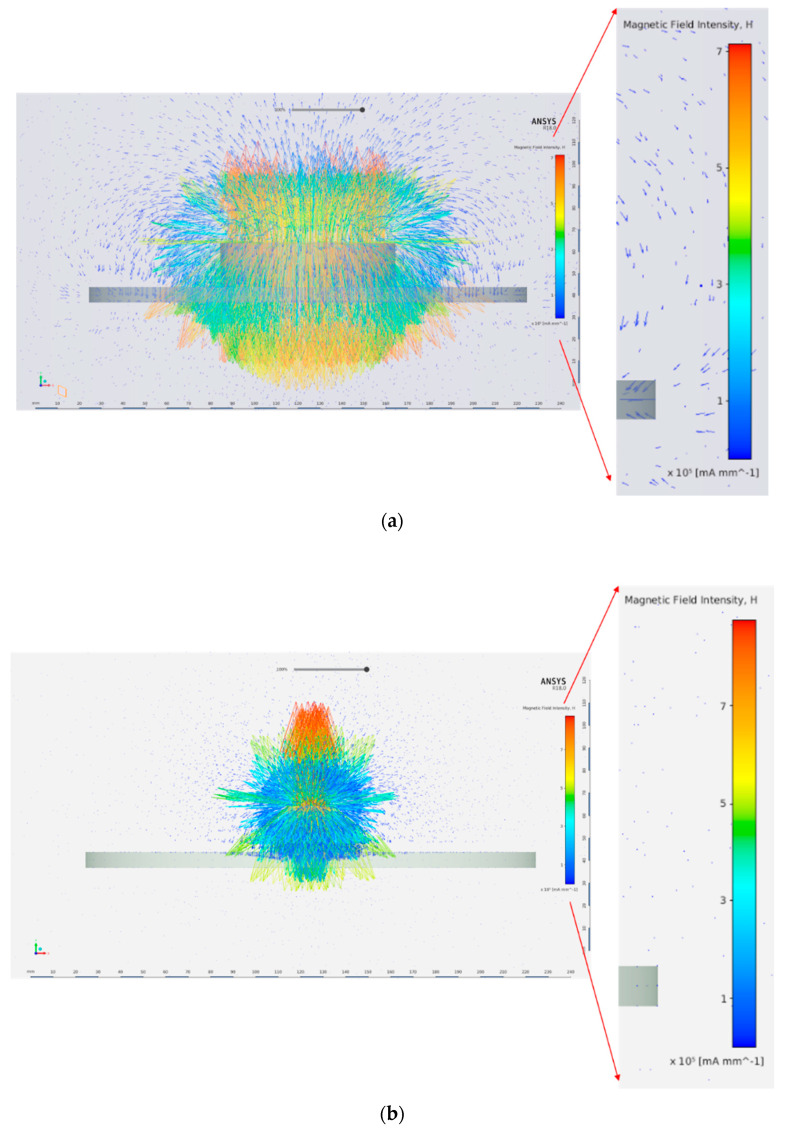
Magnetic field intensity for two magnets. (**a**) Magnet diameter = 80 mm. (**b**) Magnet diameter = 20 mm.

Furthermore, using a larger diameter magnet increases the intensity of the magnetic field lines interacting with the steel surface, which is shown in Figure 6. Figure 6 clearly shows that the magnetic field penetration through the steel disk thickness is more pronounced when the magnet diameter is larger.

#### 4.1.2. Steel Disk Surface Area

**Figure 7:**
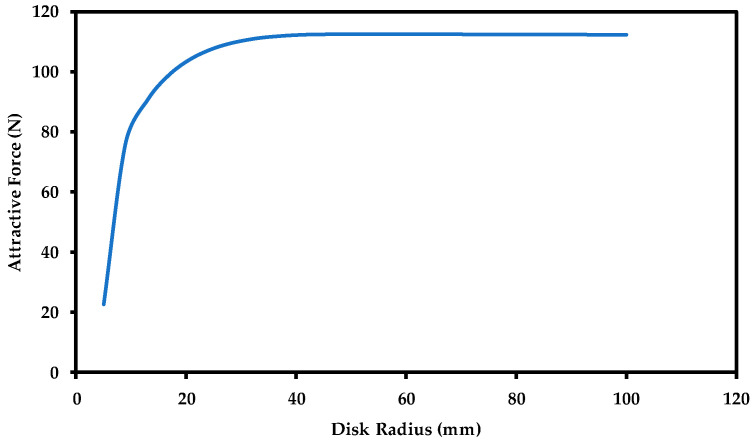
Effect of steel wall surface area on the generated magnetic force.

The steel wall, representing an oil storage tank, underneath the magnet is represented by a disk in our FE model as discussed earlier. The magnetic force generated between the magnet and the steel disk can be affected by the surface area of this disk. This relationship is represented in Figure 7. It can be noticed that the magnetic force saturates quickly as the radius (surface area) of the steel disk increases and becomes larger than the surface area of the magnet. When the steel disk radius is twice the magnet radius or larger, the attractive force remains constant. In the actual application, the surface area of the oil storage tank wall is much larger than the surface area of the magnet; so, in the FE models, as long as the steel surface area (representing the oil tank surface area) that is chosen is much larger than the magnet surface area, we will achieve proper attractive force results. In all the FE models, the steel surface area that was chosen was much larger than the magnet surface area, and it was modeled as a circular disk.

#### 4.1.3. Separation Distance (Air Gap)

**Figure 8:**
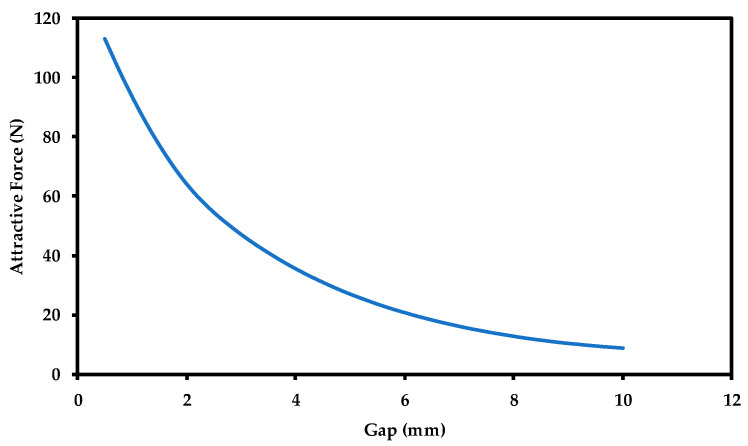
Attractive force as a function of air gap.

The separation distance (air gap) between the magnet and steel disk is one of the most important parameters that can highly impact the magnetic attractive force and magnetic field penetration through the steel disk thickness. The attractive force magnitude is very sensitive to the fluctuations of this air gap. Thus, this distance has to be carefully fixed to a specific value to avoid interfering with the generated attractive force magnitude. Figure 8 illustrates the effect of the separation distance (air gap) on the attractive force.

**Figure 9:**
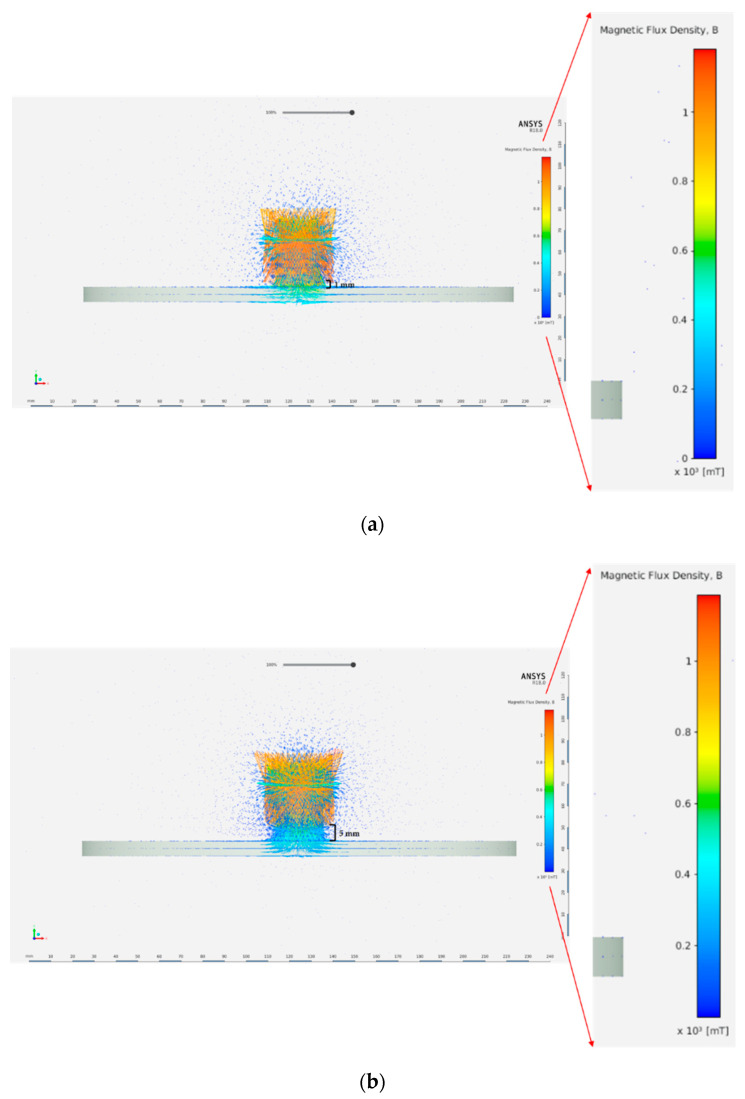
Magnetic flux density for different separation distance values. (**a**) Separation distance = 1 mm (red color indicates higher magnetic density). (**b**) Separation distance = 5 mm (red color indicates higher magnetic density).

Figure 8 shows the attractive force significantly decreasing with increase in the air gap size. The attractive force decreases by more than five times of its original value by increasing the gap size from 0.5 to 5 mm. Hence, in order to keep the attractive force and magnetic penetration through the steel thickness high, it is recommended to make the separation distance (the air gap) as small as possible. When the air gap between the magnet and the steel surface increases, the magnetic flux density, penetrating the steel surface, decreases, resulting in lower attractive force. Figure 9 shows the magnetic flux density for both cases. It can be observed that a stronger magnetic field with a larger density is penetrating the steel surface when the gap is 1 mm as compared to a gap of 5 mm.

#### 4.1.4. Steel Wall Thickness

The thickness change in the steel disk underneath the magnet represents the metal loss that occurs due to corrosion; therefore, it is the most important parameter that defines the effectiveness of our proposed sensor. The amount of force change indicates how much the thickness has changed, which depends on the ability of the magnetic flux lines to penetrate the steel surface. Using a small size magnet resulted in small magnetic penetration, thus, a low range of thickness detection.

**Figure 10:**
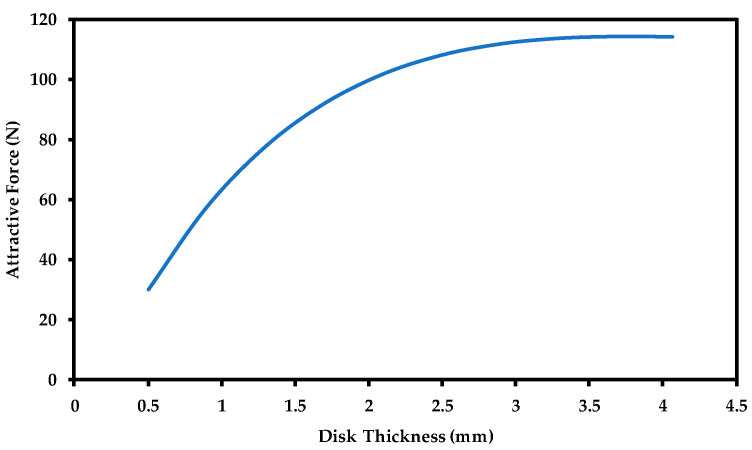
Saturation of the magnetic force when the disk thickness is increased. Air gap = 0.5 mm.

Figure 10 represents the change in the attractive force when the steel wall thickness is increased based on a 20 × 20 mm cylindrical magnet (diameter × height). The above result illustrates that a significant change in attractive force occurs at the first three millimeters of the steel disk, and after 3 mm thickness, saturation in attractive force occurs. With a 20 × 20 mm cylindrical magnet, Figure 10 implies that we will not be able to detect steel thickness change due to corrosion until the steel thickness reaches 3 mm or below.

In order to increase the thickness detection range, all the parameters that affect attractive force have to be chosen very carefully. In the following sections, an attempt was made to optimize the thickness detection range by proper selection of sensor design parameters.

## 5. Methodology and Sensor Design

Based on the FE simulation results illustrated in the previous section, each parameter analyzed has a specific impact on the attractive force magnitude. Moreover, the effect of changing each parameter on the attractive force is now known; thus, it is possible to select suitable sensor parameter values to complete the sensor design and conduct experimental tests.

It was shown that the magnet diameter has a huge impact on the magnetic force compared to the height; hence, in the corrosion detection sensor design of this paper, the rare-earth magnet diameter is chosen as three inches (7.62 cm) with a height of one inch (2.54 cm). These magnet dimensions will ensure the generation of large attractive forces without the magnet size affecting the sensor implementation in the field. Furthermore, based on our analysis, we learnt that the separation distance (air gap) between the magnet and steel disk has a significant effect on the attractive force; therefore, the gap size will be chosen as small as possible, in the order of 0.5 to 2 mm. In the experiment, the storage tank can be represented with a steel disk or a rectangular plate, as long as the disk or the rectangular steel plate surface area exceeds the magnet’s surface area.

### 5.1. Magnet Materials

Neodymium magnets are considered the most powerful rare-earth magnets available in the market. The neodymium magnet alloy is made of neodymium, iron, and boron [33]. Bonded neodymium magnets are now used in several applications such as computer peripheral, office automation, and consumer electronic applications [34]. Neodymium magnets, in comparison to regular magnets, possess higher magnetic field strength within small sizes as well as higher coercivity (the ability of the material to resist demagnetization).

The theoretical limit for neodymium magnets is N64, but commercially, neodymium rare-earth magnets available for purchase are in the range of (N35 to N52 grades), each grade exhibits different material characteristics such as magnetic field strength, coercivity, and heat tolerance. N55 and N56 rare-earth magnets are slowly being introduced to the market. Higher-grade magnets usually possess larger magnetic field strength.

The heat tolerance refers to the maximum operating temperature of the magnet; it is usually between 80 °C and 230 °C for different grades and types of neodymium magnets. In order to enhance the maximum operating temperature and corrosion resistance of neodymium magnets, they are subjected to surface treatment represented by coating the magnet with different alloys such as nickel and copper; thus, the magnet becomes more durable [35]. Due to their suitable magnetic properties, neodymium magnets are used in the corrosion sensor design of this paper. N45 material was used for our rare-earth magnet.

### 5.2. Storage Tank and Pipeline Materials

Storage tanks are made of different materials, but the most commonly used materials in storage tanks are carbon steels, stainless steels, concrete, plastic, fiberglass-reinforced plastic, nylon, and polyethylene. Oil is usually stored in vertical cylindrical tanks (twinned walled tanks with floating roofs) made of steel. Low (or mild) and medium carbon steels with carbon content of 0.05% to 0.5% are one of the essential metals that are used in all stages of the O & G industry for oil pipelines as well as oil storage tanks. The majority of oil storage tanks are made of low carbon steel, and some tanks are lined with different types of epoxy, polyurethane, or rubber in order to prevent corrosion and leakage. However, medium carbon and stainless-steel oil storage tanks (304 SS or 316 SS) are also used in the oil industry. Oil pipelines are generally made of API-5L X52-X70 material.

Ferromagnetic materials, such as carbon steel, possess a nonlinear magnetization behavior when it is subjected to a magnetic field. This response is described by the material B-H curve, which represents a nonlinear relationship between the magnetic flux density (B) and magnetic field intensity (magnetic strength, H); the ratio of these two parameters represents the material magnetic permeability.

Carbon steels have wide range of chemical compositions. For example, AISI 1020 steel has carbon content in the range of 0.17% to 0.23% or manganese, in the range of 0.3% to 0.6%; so, suppliers are allowed to make carbon steels in those ranges; thus, one can obtain all sorts of magnetic variation (B-H curves) from lot to lot, even within the same grade. In addition, there are over 3500 grades of steel, and each grade has its own alloying ingredients and carbon content percentages, thus, a different B-H curve.

Since the B-H curve of steel 1008 and 1010 exist in ANSYS, efforts were made to purchase AISI 1010 steel to represent the steel disk in our experiment (representing storage tank surface). However, low carbon steels such as AISI 1008 and 1010 are rarely available for purchase. The most readily available low carbon steels in the market are ASTM A36 or AISI 1020. AISI 1020 steel was not available to us so ASTM A36 was purchased. ASTM A36 has carbon content between 0.25% and 0.29%. We found B-H curve data for AISI 1020, and this steel has similar carbon content as A36, so we used the steel 1020 B-H curve for our ANSYS finite element analyses. A36 steel is actually close in composition to some of the steels used in the O & G industry.

### 5.3. Sensor Final Design

As explained earlier in Section 3, the sensor consists of five main parts; a rare-earth magnet encapsulated by a metal part, called the inner member, a two-piece shaft, a load cell in between lower and upper shafts, and an outer member. The magnet is securely glued to the inside of the inner member, to make sure the magnet does not move when large magnetic forces are present, and the magnet/inner member assembly is attached to the lower shaft. The other end of the lower shaft is internally threaded to attach to the load cell. The load cell is also circular and has almost the same diameter as the shafts, and it comes in between the two shafts. The shafts, the load cell, the inner member, and the magnet form an assembly, and this assembly is attached to the outer member, through an internally threaded hole in the outer member. The two-piece shafts can move up or down because of the threads. The air gap between the magnet and the steel plate is adjustable by rotating the shaft through the threaded hole. All metals components mentioned above were made of aluminum except the magnet (made of neodymium, iron, and boron), the steel plate (ASTM A36) representing the oil storage tank, and the load cell (made of stainless steel).

**Figure 11:**
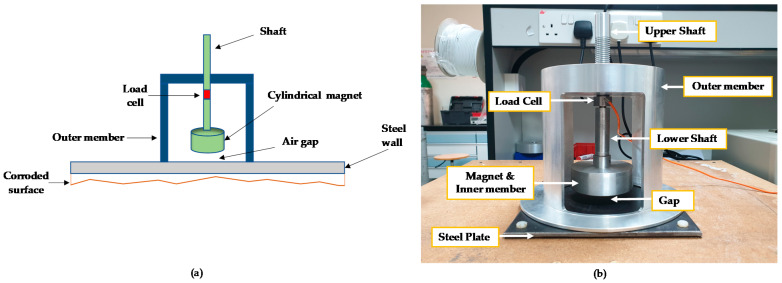
Sensor design. (**a**) Initial schematic of the sensor. (**b**) Manufactured sensor.

The attractive force, generated between the rare-earth magnet and the A36 steel plate, will place the load cell in tension, and this tension force can be measured by observing the voltage output change from the load cell. Figure 11 illustrates the schematic sensor design along with the manufactured sensor with all its components.

### 5.4. Methodology and Experimental Setup

**Figure 12:**
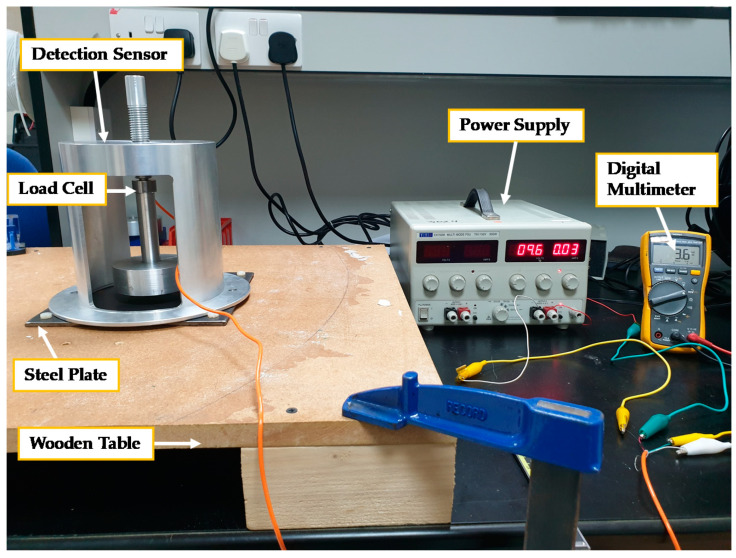
Experimental setup including all used devices.

The experimental test setup built to conduct the experiment consists of the corrosion detection sensor fully assembled with all its components, a power supply, a digital multimeter, a wooden table, plastic bolts and nuts, and rectangular A36 carbon steel plates of different thicknesses, simulating the oil storage tank. The experimental test setup was carried out using the following steps:

1. All components of the corrosion detection sensor are to be assembled first by securing the load cell between the upper and lower shafts and attaching the assembled shafts/load cell to the outer member. Finally, the magnet assembly is connected to the shafts/load cell assembly.
2. In our experiment, a steel plate was secured to a wooden table using four plastic screws/nuts. Then, the wooden table was secured to the lab bench using four C-clamps (see Figure 12). The table needs to be made of wood or any non-magnetic material, since the magnet is very powerful, and any ferromagnetic material placed near the magnet will impact magnetic flux distribution.
3. The sensor was first placed on a flat non-magnetic surface, and the shafts/load cell assembly was rotated until the desired air gap was obtained. A plastic feeler gauge was used to set the air gap. During the rotation of the shaft assembly, care needs to be taken not to cause damage to the load cell wires.
4. The corrosion sensor was then carefully placed on the steel plate, and the air gap between the magnet and the steel plate was precisely controlled for a 2nd time using a plastic feeler gauge and by small rotation of the shafts/load cell assembly. Since the rare-earth magnet has a very strong magnetic field strength, one should be very careful when placing the sensor on the steel surface.
5. The load cell comes with four wires, two input wires and two output wires. The next step was to connect the power supply to the input wires of the load cell, which represents power and ground wires. The two output wires of the load cell were connected to both terminals of the digital millimeter (DMM). The DMM should be set-up to measure voltage in millivolts (mV) with 0.001 resolution.
6. Since there is a strong attractive force between the magnet and the steel plate, a tension load will be felt by the load cell. Record the voltage associated with this attractive tension force that appears on the screen of the DMM.
7. As metal loss occurs on the steel plate due to corrosion, the magnitude of the attractive force changes over time. In our experiment, steel plates of different thicknesses were used in the experiment to simulate metal loss.
8. Experimental parametric studies can be conducted to collect attractive force data by holding the air gap fixed and changing steel plate thickness representing metal loss; or, a steel plate with a given thickness is used, but air gap is varied.

## 6. Experimental Results and Numerical Verification

The main two parameters that were experimentally studied were the effect of air gap and the steel plate thickness change on the attractive force.

### 6.1. Air Gap

From the numerical analysis conducted previously, we learnt that the developed attractive force is inversely proportional to the air gap between the magnet and the steel plate.

**Figure 13:**
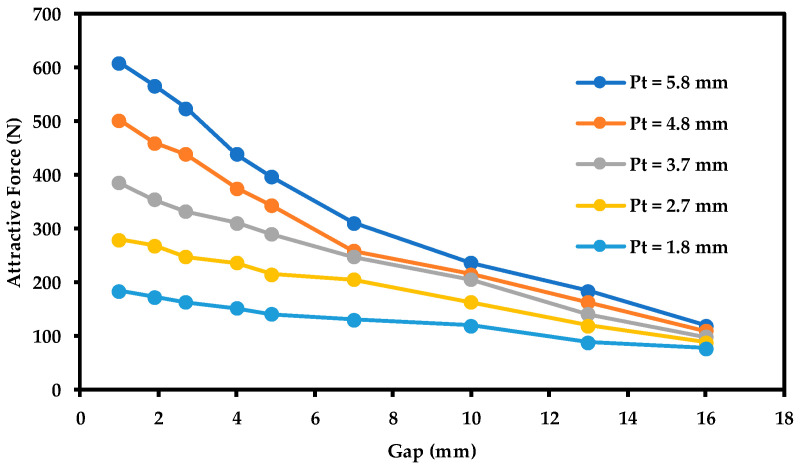
Relationship between the magnetic attractive force and the air gap between the magnet and the steel plates for different plate thicknesses. Pt refers to the steel plate thickness.

The air gap/attractive force relationship was experimentally found at several steel plate thicknesses. Figure 13 illustrates this relationship for steel plates of different thicknesses, starting from 1.8, 2.7, 3.7, 4.8, and ending at 5.8 mm.

It can be established from the experimental data of Figure 13 that the highest recorded attractive force always occurs when the air gap is 1 mm, which is the recommended separation distance for the sensor to be fixed at when it is implemented in the field. Moreover, it can be noticed that the general trend of this relationship is similar to the trend established from the numerical analysis (refer to Figure 8). In addition, from Figure 13, one can see that at higher air gaps, the change in attractive force is small as steel plate thickness changes from 1.8 to 5.8 mm. This implies that using a higher air gap will result in an earlier force saturation (smaller detection range).

### 6.2. Sensor Thickness Detection Range

**Figure 14:**
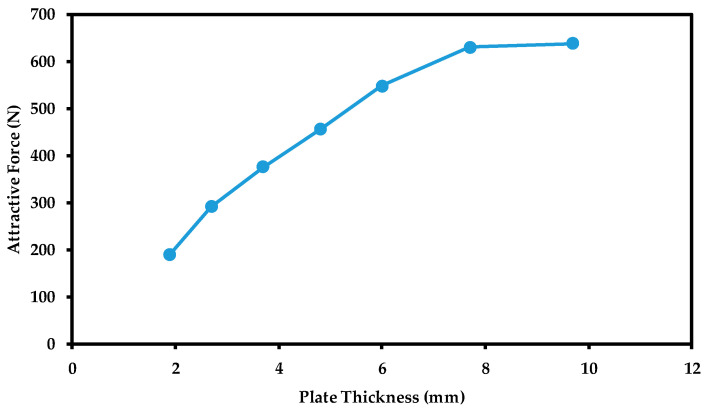
Experimental results illustrating the relationship between the magnetic force and plate thickness, which defines the thickness detection range of the sensor.

The thickness detection range of the sensor can be examined by varying the steel plate thickness with all other parameters held fixed. Different thicknesses of steel plate represent the material loss that occurs to the internal surface of the oil storage tank due to corrosion. Thus, each plate thickness represents a specific corrosion level of the internal surface. The thickness detection range is then established when the attractive force generated saturates no matter how much the thickness of the steel plate underneath the sensor is increased. Figure 14 shows the experimental attractive force data versus plate thickness using steel plates with the following thicknesses: 1.9, 2.7, 3.7, 4.8, 6.0, 7.7 and 9.7 mm with an air gap maintained at 1.5 mm. The air gap could have been fixed at a value lower than 1.5 mm, which could have enhanced the attractive force, but because the shaft threads were not fine, it was difficult to bring the air gap to lower than 1.5 mm. In future designs of our sensor, it is recommended to use finer threads on the upper shaft and the outer member to allow users to better control the air gap.

The recorded results (Figure 14) illustrate a similar trend to the data acquired by the numerical analysis (Figure 10) where the attractive force saturates upon reaching a specific disk thickness. This value of saturation defines the detection range of the sensor. It describes the thickness at which the magnetic flux lines will not be able to penetrate through the plate thickness anymore; thus, no noticeable change occurs to the attractive force. Figure 14 shows that the attractive force saturates at a thickness of 7.7 mm with a force magnitude of 636.8 N. The above data suggest that if the thickness of the storage tank is greater than 7.7 mm, our device will not be able to detect any thickness change due to corrosion until the thickness of the storage tank reaches 7.7 mm and lower.

### 6.3. Verification of Experimental Data

**Figure 15:**
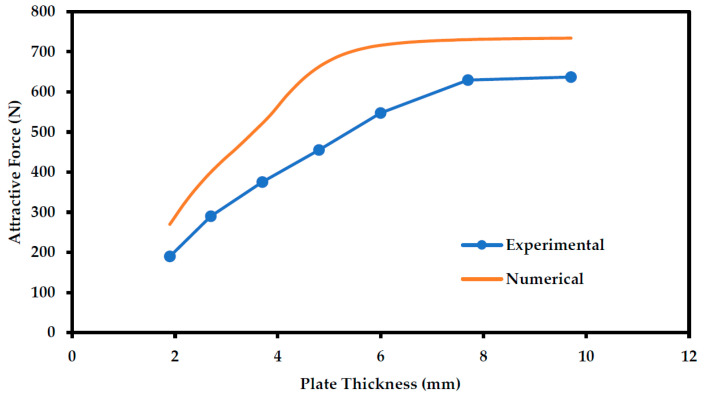
Comparison between experimental and numerical data describing the effect of plate thickness on the force when the gap is fixed at a value of approximately 1.5 mm.

In order to verify the possible thickness detection range of the sensor, a comparison between the numerical and experimental results is represented in Figure 15.

The numerical results show a similar trend with the experimental results where the relationship between the attractive force and the plate thickness is almost linear until reaching saturation. The attractive force then saturates at a specific plate thickness, which defines the corrosion detection range of the sensor. This behavior is similar for both the numerical and experimental data, but there are some differences. 

Table 1 illustrates a comparison between these two results. Even though the behavior of both results is similar, the data illustrate a difference between the experimental and numerical results, where the attractive force is supposed to saturate at a thickness of almost 6 mm according to the ANSYS simulations. Instead, the experimental analysis shows a better detection range where the saturation occurs at a higher plate thickness values, which is approximately 7.7 mm (saturation is assumed when the force difference is less than 10 N). Moreover, it is noticed that the force values recorded experimentally are generally smaller than numerical results.

As explained earlier, the steel material used to conduct the experiment was steel ASTM A36. Since the B-H curve for A36 was not available, the AISI 1020 B-H curve was used for the ANSYS analysis. The difference between experimental results and ANSYS numerical results could be due to that.

### 6.4. Discussion

The experimental results have been verified numerically by simulating the exact experimental test set-up using ANSYS finite element analysis software/an electromagnetic solver. Even though both experimental and numerical results show similar behavior and relationships between design parameters, there is always an offset between values obtained from experimental results versus numerical results. This offset in attractive force values can be explained as follows.

Firstly, it was mentioned earlier that the type of steel used in the experiment is different than the one defined in the numerical analysis. The steel used in the experiment was ASTM A36, whereas in the FE analysis, it was AISI 1020. Although the composition of both types of steel is somewhat similar, the magnetic behavior (B-H curve) may be different between the two steel types.

Secondly, the magnetic strength of the actual magnet was different from manufacturer’s published value. In the experiment, Nd_45_ magnet was used, and according to the supplier, this grade of neodymium magnets is supposed to have a maximum magnetic strength of approximately 13,500 Gauss. On the other hand, when we tested the magnet experimentally using a gaussmeter, the maximum magnetic induction (magnetic flux density) recorded was less than 5000 Gauss. This drop in the magnetic strength may have happened due to manufacturing variability, or the magnet might have experienced high temperatures during shipment. Since the value of the magnetic induction directly affects the attractive force generated between the magnet and the ferromagnetic material, it is believed that the drop in the experimental attractive force is predominantly related to the magnet magnetic strength.

Thirdly, when we conducted experiments shown in Section 5.2, every time we changed the steel plate with a new one, great efforts were made to maintain the air gap to 1.5 mm, but it is quite possible that there were variabilities in the air gap from one experiment to another.

Furthermore, multiple measurement errors may have occurred during the data collection process such as systematic errors, observational errors and/or instrumental errors, and gross errors. An observational error may have occurred due to a fault in the measurement reading and can be minimized by using a precise voltmeter; in our case, a Fluke voltmeter with a resolution of 0.001 decimals was used to minimize this error as much as possible. The instrumental error is represented by the inherent limitation of the device; in our case, the limitations of the used load cell or the effect of loading. Gross error may also have occurred throughout the experiment due to physical errors in the experiment analysis, calculations, and measurements of the experimental outcomes. These types of errors can be minimized by carefully recording the experimental data as well as recording multiple readings from the instrument.

## 7. Field Implementation

The sensor, proposed by the authors, is a discrete sensor, meaning it can only detect metal loss due to corrosion/erosion on the segment of the steel surface, which is directly beneath the permanent magnet. Metal loss away from the sensor or the magnet cannot be detected. To know the complete corrosion state of a steel surface, such as an oil storage tank bottom, several sensors need to be employed. However, using too many sensors will be costly. The best approach is to use few sensors at strategic locations on the tank bottom, with the sensors physically or wirelessly connected to a central control authority. By knowing the corrosion state of these few locations, an estimate of the corrosion state of the entire tank bottom can be made. Currently, the authors are working with another university on software that can carry out this prediction, but the details of this software are not discussed in this paper. To be able to implement the above corrosion detection sensor on oil storage tanks in the desert, electricity supply for communication and powering the load cell will be necessary. The proposed corrosion detection sensor can be used on both oil storage tanks and oil pipelines. For oil storage tanks, as explained earlier, the sensor will be a discrete sensor, meaning the sensor will require a self-contained power supply to power the load cell and the communication system. The communication system will communicate the thickness change to the control room. The electricity supply in the desert to power this sensor can be in the form of batteries or photovoltaic panels. To make the sensor completely self-contained, batteries are preferred.

Since the corrosion rate of low and medium carbon steels are on average in the range of about 1 mm per year, it is not necessary to have this corrosion detection sensor turned on continuously in real time. The sensor can automatically turn on and off or manually be turned on and off every few months, thus, the battery, if used, can last a long time. The load cell used in this paper requires a voltage of 10–12 volts (DC); so, a battery can provide this voltage easily. Lithium–ion batteries have a shelf life of 10–15 years, and they can operate in extreme temperatures, thus, they are the right choice for this application. Three AA 3.7 volts 3400 to 5000 mah lithium–ion batteries can provide the needed electricity for this sensor.

To have a wireless communication from the corrosion detection sensors to the control room, Semtech LoRa chipsets can be used. They can communicate up to 10 km and require only 4.2 mA current consumption.

Since we are dealing with flammable liquids and gases in the oil industry, extreme care needs to be taken to select the right battery, the communication system, and the overall electronics to make sure this sensor is not the cause of fires or explosions in the oil fields. Initially, it makes the most sense to operate this sensor manually, meaning an inspection engineer/technician will conduct visual inspection of the surface of the storage tank to make sure there are no oil leaks. Then, flammable/toxic gas detection sensors can be used to make sure no flammable or toxic gases are present near the oil storage tank. Once all the safety procedures are followed, then the corrosion detection sensor of this paper can be turned on for few seconds, and the load cell voltage output can be measured. A lookup table can be used to convert output voltage to thickness change.

## 8. Conclusions

An internal corrosion detection sensor for oil storage tanks and exposed oil and gas pipelines was introduced in [32] in 2018. The 2018 design was only able to detect storage tank thickness change due to corrosion when the thickness of the storage tank reached 3 mm and below.

The detection range of our proposed sensor is defined as the maximum wall thickness that the magnetic field lines can penetrate to. In the authors’ previous work [32], we were able to detect metal loss when the steel plate thickness was 3 mm or less (3 mm of magnetic field penetration), meaning if a 10 mm thick steel disk was corroding, our previous sensor design was unable to detect metal loss due to corrosion until the steel wall thickness reached 3 mm. Once steel wall thickness was 3 mm or less, our previous sensor was able to detect metal loss due to corrosion.

In the new corrosion detection sensor design of this paper, some design limitations of the 2018 design have been eliminated, and the new sensor is able to detect metal loss of steel plates due to corrosion when the steel wall thickness is 7.7 mm or less. This represents more than 2.5 times (7.7/3 mm = 2.56 times) increase in the thickness detection range as compared to our previous design. The new sensor provides an earlier warning regarding the corrosion status of the storage tank wall as compared to the old design. Additionally, in the new design, the air gap between the magnet and the steel surface can be controlled very accurately, where this was not possible with the old design.

The sensor was fully manufactured and tested experimentally, and the test data correlated very well with the FE numerical results.

In the closing remarks, it will be important to discuss few important concerns: (1) The effect of corrosion by-products remaining on the tank or pipeline surfaces and not flaking off and their impact on accuracy of the proposed sensor. (2) The accuracy of the sensor in detecting metal loss on curved surfaces. (3) The loss of magnet magnetic strength over time due to temperature and other effects. (4) The corrosion of rare-earth magnets in the field.

As for concern # 1, oil storage tanks and pipelines can corrode from outside and from inside. As for the external corrosion, depending on the environment, temperature, humidity, and other factors, the oil storage tanks and pipelines can corrode from outside, and the literature indicates that the corrosion by-products found on the external surfaces of tanks and pipelines are mostly Fe_2_O_3_, Fe_3_O_4_, or FeO(OH) or a combination of these products. As for the internal corrosion of storage tanks and pipelines, due to the presence of CO_2_, H_2_S, water, and oxygen, iron sulfides (FeS) and mackinawite (Fe_1+x_ S), iron carbonates (FeCO_3_), and iron oxide/hydroxide FeO(OH) are mostly found on the interior surfaces of storage tanks and pipelines. Other corrosion by-products have been also observed inside the tanks and pipelines, depending on the type of the crude oil, the water content, temperature, pressure, pH level, salinity, dissolved gases and their partial pressure, bacteria, and other factors.

Since the proposed sensor of this paper sits on the outer surface of tanks and pipelines, it is sensitive to both outside and inside corrosion of oil storage tanks and pipelines. Some of the corrosion by-products mentioned above are magnetic, and if these corrosion by-products do stay on the storage tank or pipeline surfaces and do not flake off, will our proposed sensor still work and detect metal loss?

In the science of magnetism, some materials are classified as ferromagnetic (strong form of ferromagnetism), some ferrimagnetic (a weak form of ferromagnetism), some paramagnetic (very weak form of ferromagnetism), and some antiferromagnetic. As for the corrosion by-products such as Fe_2_O_3_, Fe_3_O_4_, FeO(OH), FeS, and FeCO_3_, there are conflicting reports in literature on magnetic properties of these corrosion by-products. These conflicting reports make it difficult to know the interaction of our sensor with these corrosion by-products.

The latest research labels Fe_2_O_3_ and FeO(OH) as a weak form of magnetism, Fe_3_O_4_ has been labeled as both ferromagnetic and ferrimagnetic, FeS as ferrimagnetic and non-magnetic, and FeCO_3_ and mackinawite (Fe_1+x_ S) as paramagnetic. Even though some of the corrosion by-products such as iron oxides such as Fe_3_O_4_ and Fe_2_O_3_ are magnetic, they possess a very weak magnetic strength as compared to low carbon or mild steel, since the iron percentage in iron oxides is around 70% as compared to more than 98–99% for low or mild carbon steels, used to manufacture oil storage tanks and pipelines.

When an oil storage tank or pipeline starts to corrode from the outside, the chemical composition of the corroding layer (most likely Fe_2_O_3_ or Fe_3_O_4_) will change from 99% iron to 70% iron, thus, there will be a drop in attractive force, measured by the load cell. As time goes on, the oxide layer will flake off, and there will be an additional drop in the attractive force. So, the proposed sensor in this paper will indicate occurrence of corrosion even though the oxide layer may stay on the steel surface for a while. As for the inside of the oil storage tanks and pipelines, the majority of the corrosion by-products are paramagnetic, so our proposed sensor should definitely see a drop in attractive force when corrosion occurs.

As for concern # 2, curved surfaces versus flat surfaces, oil storage tanks can have corrosion at the tank bottom (bottom plates) and the tank walls. However, the most vulnerable to corrosion, in upright atmospheric aboveground cylindrical oil storage tanks, is the tank bottom. The accumulation of water at the tank bottom, since water has a higher density than the crude oil, is the primary reason why the corrosion is more severe at the tank bottom than the tank walls. So, the internal corrosion problems of most cylindrical oil storage tanks are mainly at the tank bottom, which is flat, and not on the tank walls; thus, the proposed sensor of this paper can effectively be used on the tank bottom or any flat steel surfaces to detect metal loss due to corrosion.

When curved surfaces are being monitored for corrosion such as tank walls or spherical storage tanks or pipelines, the air gap will not be the same everywhere underneath the magnet, if flat magnets are used. When there is corrosion on the tank walls of cylindrical oil storage tanks, which are curved and not flat, the proposed sensor of our paper can still be used to effectively detect tank wall metal loss due to corrosion. Most cylindrical oil storage tanks range from 10 ft (3 m) in diameter to over 412 ft (≈125 m) in diameter. The footprint area of our rare-earth magnet (3 inch in diameter magnet) is only 0.00465 m^2^. With the smallest cylindrical oil storage tanks, 3 m in diameter, which are rarely used in the oil and gas industry, analysis clearly shows that the tank walls will appear as almost flat surfaces to the magnet. For a 3-m tank, if the gap at the center of a 3-inch (7.62 cm) magnet is 1 mm, at the magnet edges, it will be 1.47 mm, meaning we should be still able to use the proposed sensor of our paper on tank bottom and walls for cylindrical tanks, without any degradation in metal loss detection range found for flat surfaces.

As for spherical storage tanks, they are used for storage of high-pressure fluids and gases. The capacity of spherical storage tanks ranges from 1000 to 75,000 barrels of oil. A spherical oil storage tank with a capacity of 1000 barrels has a diameter of 6.72 m. The calculations show that if the gap at the center of a flat 3-inch (7.62 cm) magnet is 1 mm, at the magnet edges, it will be 1.22 mm, meaning we should be able to use the proposed sensor of our paper on spherical storage tanks as well.

However, for oil and gas pipelines, which are not the focus of this paper, the air gap at the center of a flat magnet and its edges could be quite different due to the curvature of the pipeline, particularly for smaller diameter pipelines. For smaller diameter pipelines, curved rare-earth magnets are highly recommended instead of flat ones.

As for concern # 3, most rare-earth magnets do lose a percentage of their magnetic field strength over time, especially if they are subjected to temperatures that exceeds their maximum operating temperature or strong demagnetizing fields. For this project, neodymium magnets were intentionally chosen, since they have a very high coercivity (the ability of the material to resist demagnetization) as compared to other permanent magnets, and they can operate in a wide temperature range that can reach up to 230 C. Neodymium magnets with suffix “AH” can operate until 230 C. [35].

An article [36] suggests that a neodymium magnet loses approximately 5% of its magnetism every 100 years. Others have indicated that they lose less than 1% of their strength over a period of 10 years. Most oil pipelines are designed for a 30- to 50-year life span, but there have been oil pipelines lasting 65 years. As for oil storage tanks, their life span is usually around 20 years. Given the fact that change in magnetism of rare-earth magnets is only 5% over 100 years, there is little concern for the magnets stop functioning over the lifespan of oil storage tanks and pipelines. As for oil storage tanks, since their lifespans are only 20 years, our proposed sensor should function well during this period.

As for concern # 4, the corrosion of rare-earth magnets are more of a concern than the loss of magnetic field strength in the oil field, but luckily, rare-earth magnets can be coated with PTFE, Ni-Cu-Ni, or Ni-Cu-Ni-Au, and other coatings to increase their resistance to corrosion.

The sensor proposed in this paper can be further improved if the overall size of the sensor can be reduced without a loss in the metal loss detection range. Another area of improvement would be to increase the metal loss detection range beyond 7.7 mm. Another possible area of improvement is to limit the number of sensors to only few, thus, reducing sensor cost, but design the sensors as such so that they can move and check for metal loss on the surface of oil storage tanks and pipelines, autonomously.

## Figures and Tables

**Table 1:** Sensor detection range acquired experimentally vs. numerically.

Plate Thickness (mm)	Attractive Force (N)	
	Experimental	Numerical
1.9	189.6	257.9
2.7	290.5	377.5
3.7	375.5	491.3
4.8	455.2	614
6	547.6	655.4
7.7	629.4	665
9.7	636.8	669.1

## Data Availability

The data presented in this study are available on request from the corresponding author. The data presented in this study can also be found from MSc thesis of Ahmad Aljarah, from Khalifa University’s Institutional Repository database.
